# Supplementary material for: Genetic encoding of a highly photostable, long lifetime fluorescent amino acid for imaging in mammalian cells
Source: Chem Sci. 2021 Aug 3;12(36):11955–64. doi: 10.1039/d1sc01914g (PMC8634729; doi:10.1039/d1sc01914g)
Supplement: SC-012-D1SC01914G-s013 [file SC-012-D1SC01914G-s013.pdf]

Computational Modeling Protocols

§1- Preparation of starting PylRS structure

PDB 2ZIN was obtained from the PDB. The Boc-Lys ligand was manually changed to Ala in PyMol using the Mutagenesis Wizard. The structure (`2zin_edit.pdb`) was prepared for Rosetta modeling:

```
/Applications/rosetta_bin_mac_2018.33.60351_bundle/main/source/build/src/release/macos/10.13/64/x86
/clang/9.0/static/score_jd2.static.macosclangrelease -renumber_pdb -ignore_unrecognized_res -s
2zin_edit.pdb -out:pdb
```

To model missing loops into the prepared structure (`PreppedRS.pdb`), a blueprint file was generated:

```
/Applications/rosetta_bin_mac_2018.33.60351_bundle/tools/remodel/getBluePrintFromCoords.pl  -pdbfile
PreppedRS.pdb -chain A>MmPylRS.remodel
```

The blueprint was modified such the rotamer, identity, and secondary of all residues solved in the structure would remain fixed. The identity of missing residues was defined and the secondary structure of them and those residues immediately up and downstream was defined as loop. An excerpt illustrating this is shown below:

```
18 K . NATRO NATAA
19 D . NATRO NATAA
20 E L NATRO NATAA
0 X L PIKAA I
0 X L PIKAA S
0 X L PIKAA L
0 X L PIKAA N
21 S L NATRO NATAA
22 G . NATRO NATAA
23 K . NATRO NATAA
```

In this segment, a four-residue loop (ISLN) is missing from the structure. Its secondary structure and that of the residues immediately up and downstream was defined as loop (L).

The identities of all residues included in the structure were kept constant (NATAA).

The rotamer orientations of all residues included in the structure were kept constant (NATRO).

After finalizing the blueprint file (`MmPylRS.remodel`), a RosettaScripts xml file was written containing the RemodelMover, which was then used to build in missing segments as follows:

```
/Applications/rosetta_bin_mac_2018.33.60351_bundle/main/source/bin/rosetta_scripts.static.macosclan
grelease -s PreppedRS.pdb -parser:protocol RemodelMover.xml -run:chain A -overwrite -score:weights
beta_nov16_cart  -corrections:beta  True  -packing:use_input_sc  -packing:ex1  -packing:ex2  -
remodel:num_trajectory 10 -remodel:use_blueprint_sequence -remodel:save_top 1
```

The structure (`PreppedRS_Remodel.pdb`) was then renumbered for further modeling:

```
/Applications/rosetta_bin_mac_2018.33.60351_bundle/main/source/build/src/release/macos/10.13/64/x86
/clang/9.0/static/score_jd2.static.macosclangrelease -renumber_pdb -ignore_unrecognized_res -s
PreppedRS_Remodel.pdb -out:pdb
```

Finally, this structure (`PreppedRS_Remodel_ReNum.pdb`) was relaxed into the beta_nov16 scorefunction:

```
/Applications/rosetta_bin_mac_2018.33.60351_bundle/main/source/bin/relax.static.macosclangrelease -s
PreppedRS_Remodel_ReNum.pdb -overwrite -database
/Applications/rosetta_bin_mac_2018.33.60351_bundle/main/database/ -score:weights beta_nov16_cart -
corrections:beta_nov16 True -flip_HNQ -no_optH false -dunbrack_prob_buried 1 -
dunbrack_prob_nonburied 1 -packing:use_input_sc -packing:ex1 -packing:ex2 -
relax:constrain_relax_to_start_coords -relax:ramp_constraints false -relax:minimize_bond_angles -
relax:dualspace True -nonideal -relax:thorough True
```

The resulting structure (`Relax01.pdb`) was then used for modeling of enzyme mutations.

§2- Modeling of PylRS variants 41 & 82

PylRS variants were modeled in PyRosetta4 (Mac version 203 for Python 3.6). A script was written which first mutated all relevant residues to their mutant identities. The pose was then FastRelaxed with coordinates constrained to using the following settings:

```
relax=pyrosetta.rosetta.protocols.relax.FastRelax()
relax.dualspace(True)
relax.minimize_bond_angles(True)
relax.set_native_pose(nativepose)
relax.constrain_coords(True)
relax.constrain_relax_to_native_coords(True)
relax.ramp_down_constraints(False)
relax.set_scorefxn(scorefunction_cart)
relax.set_movemap_factory(relax_movemapfactory)
relax.set_task_factory(relax_taskfactory)
```

The relax_taskfactory included all residues with the IncludeCurrent(), RestrictToRepacking(), and InitializeFromCommandLine() task operations with the following CommandLine imported at the start of the script from a txt file:

```
-score:weights beta_nov16_cart
 -corrections:beta_nov16 true
 -in:file:native InputStructures/PreppedRS_Remodel_ReNum.pdb
 -relax:constrain_relax_to_native_coords true
 -packing:ex1
 -packing:ex2
 -packing:use_input_sc
 -packing:dunbrack_prob_buried 1
 -packing:dunbrack_prob_nonburied 1
 -shapovalov_lib_fixes_enable True
 -mute all
 -nonideal
```

The relax_movemapfactory allowed all chi angles, backbone, bond angles, and jumps to move and prohibited bond lengths from moving.

Structures were then saved to PDB files for both enzyme 41 (`Relaxed_41mutations_withlig.pdb`) and enzyme 82 (`Relaxed_82mutations_withlig.pdb`).

Then, the Ala ligand for each was deleted and ligand-free structures were output to PDB files (`Relaxed_41mutations.pdb` & `Relaxed_82mutations.pdb`).

§3- Docking of Acd into PylRS variants 41 & 82

The following docking protocol was repeated 3 separate times from start-to-finish for enzyme 82.

To initially position the Acd ligand in the binding pocket, DARC was used.[1] Ray files were generated for the pocket surrounding the polar mutations at positions 311 & 382 (positions 159 & 230 in the model). Rays were cast using the Ala ligand from `Relaxed_82mutations_withlig.pdb` as the origin:

```
/Applications/rosetta_bin_mac_2018.33.60351_bundle/main/source/bin/make_ray_files.static.macosclang
release -database /Applications/rosetta_bin_mac_2018.33.60351_bundle/main/database/ -protein
Relaxed_82mutations_withlig.pdb -central_relax_pdb_num 159:A,230:A -set_origin 5 -multiple_origin -
origin_res_num 268:B -darc_shape_only true -pocket_static_grid true -pocket_num_angles 100 -
pocket_grid_spacing 0.5 -pocket_probe_radius 1 -pocket_max_spacing 25 -pocket_surface_dist 2.5 -
pocket_filter_by_exemplar true -overwrite
```

A params file and rotamer library for ACD were generated using a previously published method.[2] A PDB file containing only Acd (`LIG.pdb`) was modified such that ACD was now called LIG. A copy of the params file (`LIG.params`) was then edited such that ACD was named LIG and the PROTEIN and L_AA properties were removed from the properties list. DARC was performed using:

```
/Applications/rosetta_bin_mac_2018.33.60351_bundle/main/source/bin/DARC.static.macosclangrelease -
protein Relaxed_82mutations.pdb -ligand LIG.pdb -extra_res_fa LIG.params -ray_file
ray_Relaxed_82mutations_withlig_159:A,230:A.txt -darc_shape_only -score:weights beta_nov16 -
corrections:beta_nov16 True -num_runs 500 -num_particles 500
```

The positioned ligand pdb output was edited so that the LIG name was changed back to ACD. The LIG residue from the pdb output of the enzyme/ligand complex was replaced with ACD and the merged pdb (`DARC_82_merge.params`) was prepared for further modeling:

```
/Applications/rosetta_bin_mac_2018.33.60351_bundle/main/source/build/src/release/macos/10.13/64/x86
/clang/9.0/static/score_jd2.static.macosclangrelease -renumber_pdb -ignore_unrecognized_res -s
DARC_82_merge.pdb -out:pdb
```

Finally, the position of Acd in the pocket was optimized in PyRosetta using the `beta_nov16`. Firstly, the ligand position was adjusted. A FastRelax of the ligand constrained to starting coordinates without ramping-down was performed in DualSpace, allowing chi angles, backbone, bond angles, and jumps to minimize only at the ligand residue (i.e., the enzyme remained unmodified).

Next, the ligand and all enzyme residues within 10Å of it were packed using the PackRotamersMover and the `beta_nov16_soft` scorefunction. The full structure was then minimized in Cartesian space using the `beta_nov16_cart` scorefunction and the MinMover.

Finally, the full structure was relaxed as above via FastRelax two times sequentially. The first time it was constrained to starting coordinates with ramping down of constraints. The second time all constraints were removed.

NB- due to steric constriction of the pocket, Acd could not be initially positioned well in the pocket of enzyme 41 using DARC (see **Fig. 1**). Therefore, to model enzyme 41, all steps of the docking protocol following DARC were performed after changing C313G back to C313 in the fully docked 82 structure.

§4- Analysis of Acd docking

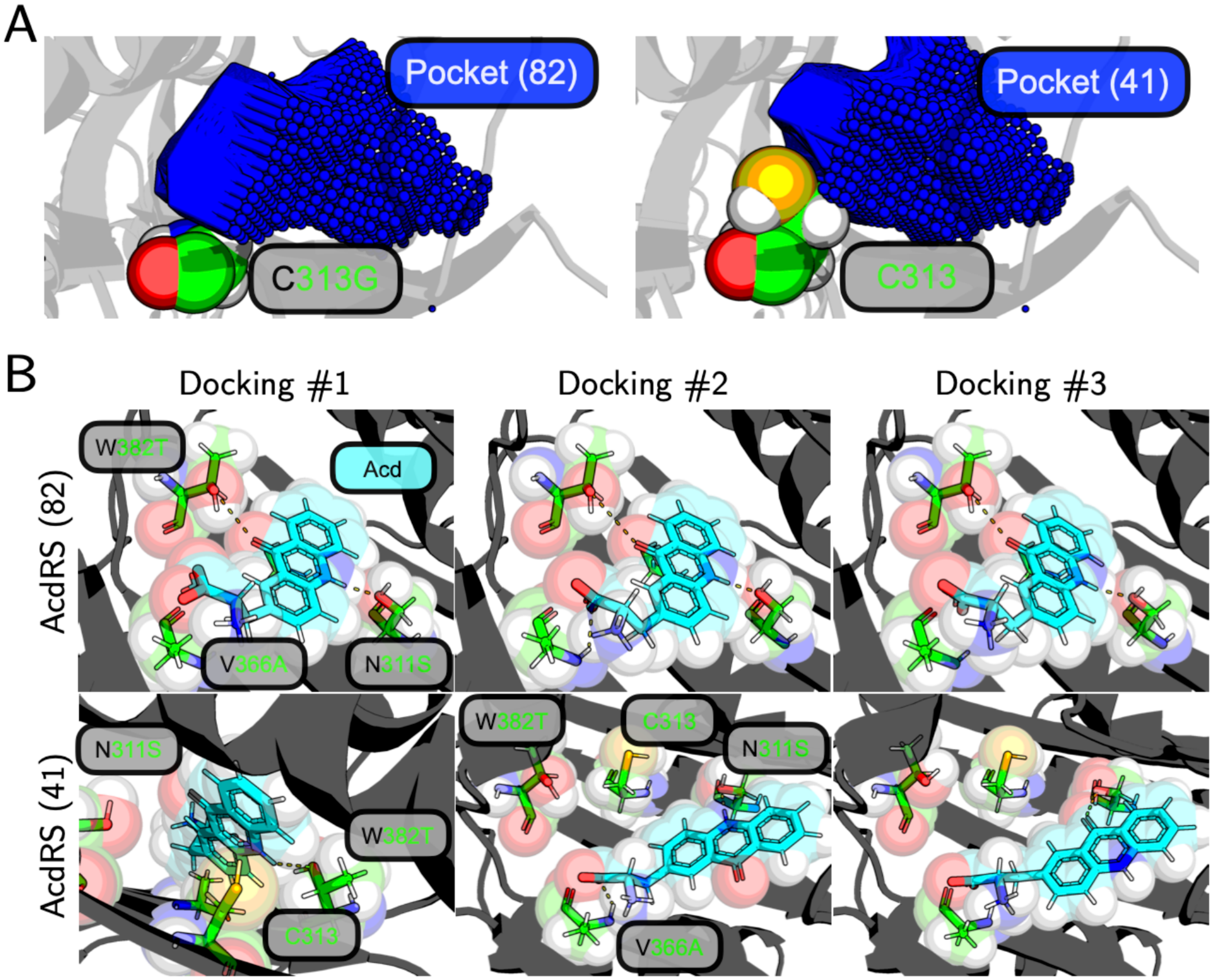

Fig. 1. A) The C313G mutation opens up extra space in the binding pocket of AcdRS (82) as compared to AcdRS (41). For this reason, Acd could be positioned well in the pocket of (82) but not of (41) with DARC. **B)** Three full docking runs were performed for AcdRS (82) and then for AcdRS(41) starting with the (82) structures. In all cases, Acd was positioned in the (82) pocket such that it could make hydrogen bonds with both W382T and with N311S. This was enabled by the steric relief from the C313G mutation. On the other hand, Acd adopted a variety of states in the (41) pocket, the first of which is productive for tRNA aminoacylation. In this state, Acd acts as a donor to the W382T residue. Note, it is unable to interact with N311S due to the steric clash from C313.

References

1. R. Gowthaman, S. Lyskov and J. Karanicolas, *PLOS ONE*, 2015, **10**, e0131612.

2. K. Drew, P. D. Renfrew, T. W. Craven, G. L. Butterfoss, F.-C. Chou, S. Lyskov, B. N. Bullock, A. Watkins, J. W. Labonte, M. Pacella, K. P. Kilambi, A. Leaver-Fay, B. Kuhlman, J. J. Gray, P. Bradley, K. Kirshenbaum, P. S. Arora, R. Das and R. Bonneau, *PLOS ONE*, 2013, **8**, e67051.
