# Supplementary material for: Genetic encoding of a highly photostable, long lifetime fluorescent amino acid for imaging in mammalian cells
Source: Chem Sci. 2021 Aug 3;12(36):11955–64. doi: 10.1039/d1sc01914g (PMC8634729; doi:10.1039/d1sc01914g)
Supplement: SC-012-D1SC01914G-s014 [file SC-012-D1SC01914G-s014.docx]

**Contents**

Fig. S1-S9: Primary data for photophysical measurements are provided as tables in Microsoft Excel files (.xlsx) or along with fitting in Graphpad Prism files (.pzfx), named according to the corresponding ESI figure.

Fig. S10: Screening data for synthetase mutants in *E. coli* shown in ESI Fig. S10 are given as a table in a Microsoft Excel file (.xlsx).

Fig. 2 and Fig. S11: Atomic coordinates for AcdRS 41 and 82 models are provided as .pdb files.

Computational modeling protocols, including code segments, are provided in a pdf document.
